# New Animal Models for Understanding FMRP Functions and FXS Pathology

**DOI:** 10.3390/cells11101628

**Published:** 2022-05-12

**Authors:** Eliza Curnow, Yuan Wang

**Affiliations:** 1 REI Division, Department of ObGyn, University of Washington, Seattle, WA 98195, USA; 2 Washington National Primate Research Center, University of Washington, Seattle, WA 98195, USA; 3 Program in Neuroscience, Department of Biomedical Sciences, College of Medicine, Florida State University, Tallahassee, FL 32306, USA

**Keywords:** nonhuman primate, Mongolian gerbil, chicken embryo, sensory deficits, brain development, social communication, gene editing

## Abstract

Fragile X encompasses a range of genetic conditions, all of which result as a function of changes within the *FMR1* gene and abnormal production and/or expression of the *FMR1* gene products. Individuals with Fragile X syndrome (FXS), the most common heritable form of intellectual disability, have a full-mutation sequence (>200 CGG repeats) which brings about transcriptional silencing of *FMR1* and loss of FMR protein (FMRP). Despite considerable progress in our understanding of FXS, safe, effective, and reliable treatments that either prevent or reduce the severity of the FXS phenotype have not been approved. While current FXS animal models contribute their own unique understanding to the molecular, cellular, physiological, and behavioral deficits associated with FXS, no single animal model is able to fully recreate the FXS phenotype. This review will describe the status and rationale in the development, validation, and utility of three emerging animal model systems for FXS, namely the nonhuman primate (NHP), Mongolian gerbil, and chicken. These developing animal models will provide a sophisticated resource in which the deficits in complex functions of perception, action, and cognition in the human disorder are accurately reflected and aid in the successful translation of novel therapeutics and interventions to the clinic setting.

## 1. Introduction

Fragile X syndrome (FXS), the most common form of heritable intellectual disability, is caused by a disruption of the fragile X mental retardation 1 gene (*FMR1*; also called fragile X messenger ribonucleoprotein 1 gene) on the X chromosome and the resultant absence of FMR protein (FMRP) [1,2]. For the most part, the loss of FMRP is caused by the expansion of the trinucleotide CGG repeat in the 5′ UTR of the *FMR1* gene [3]. Loss of FMRP results in the characteristic features of FXS, including mild to severe intellectual disability, cognitive impairments, seizures, poor language development, altered physical features such as macroorchidism and facial dysmorphisms, and behavioral problems such as social difficulties, anxiety, hyperactivity, hypersensitivity to sensory stimuli, and other autistic-like behaviors [4,5,6,7,8]. Behavioral deficits in FXS can be detected as early as infancy and toddlerhood and can last a lifetime (reviewed in [9]).

There are no known naturally occurring animal models of Fragile X disorders. Current animal models of FXS, both invertebrate (*Drosophila*, fruit fly [10,11,12]) and vertebrate (*Mus*, mouse; *Rattus*, rat; *Danio*, zebrafish [13,14,15,16,17,18,19]), have focused on loss-of-function models with disruption or knockout (KO) of the *FMR1* gene homolog. Given the degree of brain homology between mouse and human, the mouse model has provided valuable insight into regional neuropathological effects resulting from a loss of FMRP. Using established test batteries, the mouse and more recently the rat have also informed our understanding of the learning, motor, cognitive, and behavioral deficits associated with FXS [20,21,22]. However, results from FXS rodent models have been inconsistent [14,17,23,24], perhaps due to the challenges of modeling higher cognitive functions and social behaviors in these species and limitations specific to sensory, biochemical, and anatomical differences between humans and rodents. The fruit fly has a single *FMR1* gene homolog (*dFmr1*) that has allowed for the development of several loss-of-function mutations to model FXS [10,12]. The homozygous mutants display abnormal behavior along with altered synaptogenesis and spermatogenesis, which may in part resemble the human FXS phenotype [25,26,27]. The strength of the fly model lies in its high-throughput genetic and pharmacological screening capabilities and accompanying lower costs and greater speed in identifying novel pathways and processes that contribute to or protect against FXS. However, the limited homology between fly and human proteins means that candidate molecules and pathways identified in the fly model ultimately require further testing in a mammalian model of FXS before translation to clinical trials. More recently, a new vertebrate model system with which to study FXS has been generated: *Fmr1* KO zebrafish [15,19]. Similar to the fruit fly, zebrafish models are of interest because they have the potential to contribute significantly to high-throughput drug screening and rescue studies [28,29,30]. However, the zebrafish does not possess the complex behavioral phenotypes found in higher order mammals.

The existing FXS animal models each contribute a unique understanding to the molecular, cellular, physiological, and behavioral deficits associated with FXS. In particular, the mouse and fly models have been instrumental in the identification of several molecular mechanisms that may underlie this disorder [31,32,33,34,35]. As a result, several targeted drug therapies have been tested in clinical trials [36,37,38,39] and while some have shown improvement in clinical outcomes, there remains a need for safe and effective treatments for FXS individuals. With the limited availability of FXS patients for enrollment and the cost and time associated with clinical trials, animal models will continue to play an important role in identifying and testing candidate compounds with therapeutic promise. This review explores the potential of new animal model systems that are in development to support and promote basic, translational, and clinical studies of FXS.

## 2. Strategies and Current Status in the Development of a Nonhuman Primate Model of FXS

Developing a nonhuman primate (NHP) model of FXS requires a systematic and strategic approach to meet the challenges of ethics, cost, and timeline that typifies research involving NHPs. However, efforts to meet these challenges are worthwhile given the translational value that the NHP represents to substantially address the limitations encountered with current model systems. An NHP model would contribute significantly to the understanding of higher cognitive functions controlled by the prefrontal cortex [40], complex social behaviors that incorporate visual and auditory cues [41,42], neurotransmitter and neuromodulatory systems [43], brain connectivity where cell type and size are important [44,45], and neurodevelopmental timelines [46,47] associated with early learning, puberty, and the aging process in FXS. Here, we outline two key strategies for the development of NHP disease models and the current status of NHP models for FXS.

Historically, gene editing in macaques has been laborious and inefficient. While notable successes with virus-mediated transgenesis have produced disease models including Huntington and Rett disease in the NHP, limitations due to the random insertion of size-limited exogenous genes coupled with very low efficiency in live birth outcomes hampered initial efforts [48,49,50,51]. With the advent of engineered nucleases such as TALEN and CRISPR/Cas9, efficient point or indel mutation of endogenous genes has greatly expanded the possibility for generating NHP models of human disease. Although these new tools are more promising in terms of NHP model fidelity and efficiency, consideration for unique requirements such as housing, specialized embryology skills, and experienced care for offspring and their long-term maintenance remain and necessitate that these endeavors are undertaken at established and accredited primate centers.

Ideally, the production of a new animal model should strive for high construct validity to recapitulate the human genetic condition. Of note, only apes—a subset of old-world primates that includes the macaque—and the squirrel monkey, a new world primate, have been identified as having *FMR1* CGG repeat lengths within the normal human range (23–45 repeats), although these are differentiated in old-world primates by one to three single G interruptions rather than the AGG observed in the human [52,53,54,55]. In the context of FXS, high construct validity would either require the insertion of an expanded CGG repeat under *FMR1’*s endogenous promoter to cause its hypermethylation and inactivation or destabilization of the endogenous repeat tract perhaps through removal of the ‘G’ interruption/s to permit in situ CGG repeat expansion. Under these conditions, the NHP FXS model would reveal to a greater extent interaction of the expanding/ed repeat with a toxic gain-of-function role and associated loss of FMRP. It has so far been proven to be technically difficult to manipulate long, pure CGG repeats for transgenic purposes. While a full mutation knock-in has not been successful and our own efforts to knock-in a human pre-mutation construct (CGG_99_) into macaque ESCs has thus far been unsuccessful, a premutation knock-in mouse model has been achieved. This mouse model has provided critical insight into repeat instability and expansion mechanisms, although subsequent full mutation length (>200) CGG repeats have not demonstrated the expected hypermethylation or gene silencing of *FMR1* [56,57]. In the human, *FMR1* gene deletions, promoter variants, missense, and nonsense mutations also result in an FXS phenotype [58,59,60,61] and formed the basis of a recent knock-in mouse that offers a new preclinical model for testing FXS drug candidates [62].

**Figure 1:**
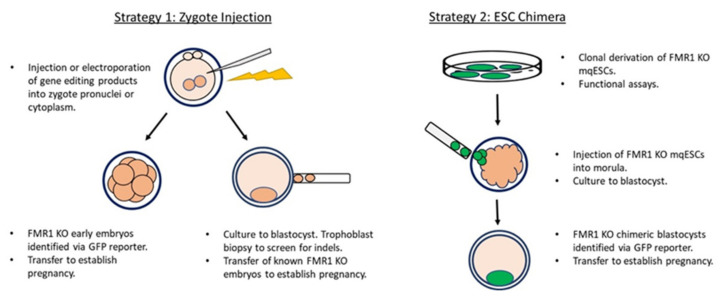
Strategies for modeling Fragile X syndrome in the nonhuman primate.

Currently, the best approach to developing an animal model of FXS continues to involve a loss-of-function strategy. We propose two main strategies for developing an NHP model of FXS that considers the practicalities and limitations as well as the ethics for working with this species. The first strategy (Strategy 1: Figure 1) involves zygote injection: Gene editing products are injected either into the pronucleus or cytoplasm of the embryo shortly after fertilization. This approach mirrors techniques used extensively in mouse model development, in which animal numbers and short generational intervals permit the selection and expansion of the desired genotype/phenotype [63,64,65]. Delivery of engineered nucleases into zygotes by electroporation has been tested in a range of species. It provides a faster and technically easier option that has reported high rates of embryo survival and targeting efficiency with a reduced incidence of mosaicism [66,67,68]. For application to the NHP, the strategy should also include a plan to identify or select mutant embryos prior to performing embryo transfer into recipient females. This allows a reduction in recipient number and avoids production of wild-type infants or infants that harbor an unsuitable or mosaic mutation. Use of a fluorescent reporter would permit some assurance that early embryos carry the mutation while trophoblast biopsy at the blastocyst stage would permit screening and sequencing of a mutation prior to transfer. Inclusion of embryo sex determination would also allow identification of female and male embryos and an opportunity to assess allele heterozygosity and mosaicism [68,69,70]. Efforts to reduce the possibility of somatic mosaicism, which could complicate subsequent studies, should be made to ensure efficient utilization of the NHP resource Additionally, establishing pregnancies with embryos of a known *FMR1* genotype provides a basis for including measures of gestational morphometrics and establishment of FXS-related pregnancy morphometrics that may contribute further to the identification of physical and possibly molecular biomarkers as this model system develops.

An alternate strategy (Strategy 2; Figure 1), which has been highly successful in mice [71,72], is the embryonic stem cell (ESC)-embryo complementation approach for the formation of chimeras. This approach has the benefit of identifying and validating a specific *FMR1* mutation in a macaque ESC (mqESC) system prior to its use in generating a whole animal model. Early efforts to produce ESC-embryo chimeras in the NHP using blastocyst complementation were unsuccessful [73], possibly due to inherent differences in blastocyst development between mouse and primate embryos [74,75]. However, aggregation of naive and primed-state mqESCs with morula stage embryos did result in chimeric fetuses with contribution of the mqESCs across all three germ lineages [76] With further refinements in methodology, live born ESC-chimeric macaques have now been produced with evidence of germline transmission [77]. With additional improvements in in vitro culture conditions and understanding of differing states of primate stem cell pluripotency, the generation of high-contribution and germline ESC-chimeras in the NHP may soon be realized [78,79,80].

Exploring both strategies, we designed TALENs to introduce mutations within regions known to cause the FXS phenotype in the highly conserved residues of the K-homology domains [81,82,83,84]. The KH0 domain in Exon 6 and the KH1/KH2 domain in Exon 9 are domains largely responsible for FMRP’s ability to bind mRNA and associate with polyribosomes [58]. In the human, frameshift and missense mutations in or near these regions have resulted in moderate to severe FXS phenotype in the absence of an expanded trinucleotide repeat sequence [81,82]. When tested in mqESCs, mutation rates ranged from 0.41 to 0.61 for Exons 6 and 9, respectively, with deletions from 2 to >750 bp. We observed >75% loss of FMRP with a concomitant reduction in *FMR1* expression in these KO mqESCs. Interestingly, we also observed in some KO mqESC lines truncated FMR proteins with very low/null full length FMRP. While there are some FXS individuals that present with *FMR1* variants that result in truncated FMRP [60,85], targeting the *FMR1* promoter and Exon 1 and 2 regions in the macaque to generate a true null phenotype may prove a better strategy. When we injected these same TALENs directly into macaque zygotes, the embryos produced showed loss of FMRP expression.

Recently, CRIPSR/Cas9 technology was employed to generate a marmoset model of FXS [86]. In this study, cytoplasmic injection of gRNA targeting the *FMR1* coding region resulted in six newborns from 27 transferred embryos carrying deletions ranging from 1 to 21 bp. Unfortunately, only one infant, notable for a mosaic genotype, survived beyond 8 days. Germline transmission of the *FMR1* mutant allele (15 bp deletion) has been confirmed in this male, representing the first founder animal for a NHP model of FXS. In human FXS pregnancies, *FMR1* remains transcriptionally active during early embryonic development before becoming silenced, although differences in the timing of gene methylation in fetal and chorionic villi are noted [87,88]. This distinction may be important in higher mammals/primates versus rodents and may be implicated in the lethal phenotype observed in the marmoset FMR1 KO model.

Whichever approach is taken, the primary objective is to establish germline transmission of the mutant allele, characterize the founder population, and generate high-value FXS infants. Consideration for the care of FMR1 KO infants, particularly those with a severe phenotype, is necessary and facilities should be prepared to provide experienced nursery and veterinary care for these potentially medically fragile infants. Under standard housing conditions, NHP infants are born and raised under the care of their mother, usually in a group setting. While it is unclear from the marmoset outcomes if the reported outcomes were a direct or indirect result of the FMR1 KO [86], there remains a paucity of information about how an NHP FXS model will present. At birth NHP infants, like humans, are completely dependent on maternal care and it is conceivable that NHP FXS infants may have deficits that could impact their ability to cling at birth, vocalize or demonstrate appropriate auditory responses or thrive due to gastrointestinal issues [89]. Furthermore, if seizure activity is present, timely and effective interventions may be necessary but difficult to administer in the setting of maternal rearing. Additionally, while infanticide amongst primates is uncommon, infant abandonment or abuse can occur, particularly with young or first-time mothers [90]. Until there is a clear understanding of the physical and behavioral phenotype in NHP FXS infants, a conservative approach would be to employ nursery or hand-rearing to successfully raise and socialize infants [91].

The NHP has the potential to provide a sophisticated model in which deficits in higher cognitive function and behavior can be associated with the loss of FMRP to accurately reflect the human disorder. The similarities between the NHP and human brain with respect to anatomy and size facilitates studies involving noninvasive imaging modalities of FXS pathology, while longitudinal studies would provide a high impact system in which to study deficits in complex functions of perception, action, and cognition associated with FXS. New and targeted therapies would also benefit because an NHP model can best mimic the neurological, endocrine, and metabolic processes of the human and facilitate preclinical studies with multi-target approaches. Similarly, the endo- and behavioral phenotype and associated age-dependent effects of FXS would be ideally suited to the NHP model. With better access to very early postnatal time points, studies aimed at early detection as well as early intervention strategies can also be undertaken. Additionally, the longer lifespan of the NHP allows for the inclusion of longitudinal studies in which to characterize the dynamic nature of FXS and potentially identify moderators or mediators of the phenotype. Finally, identification of novel cellular and molecular mechanisms may prove to be useful targets for intervention in FXS as well as other autism-related disorders in the human, such as Rett syndrome and Angelman syndrome, which similarly involve deficits in higher cognitive function and abnormal behaviors.

## 3. The Mongolian Gerbil Is a More Human-like Rodent Species for Modeling Sensory Dysfunction and Social Difficulties in FXS as Compared to Mice and Rats

A fundamental function of the brain is to process, integrate, and interpret sensory information for decision making. Sensation emerges before birth and early in life, playing important roles in the construction, maturation, maintenance, and plasticity of brain network systems. Sensory deficits are a hallmark feature of many types of neurodevelopmental disorders including FXS [92,93]. Given that vertebrate species including humans share general organization of sensory systems, several rodent species, particularly mice, rats, and gerbils, are used as model organisms for understanding sensory processing mechanisms and sensory–cognition interactions under both physiological and pathological conditions [18,94,95,96,97]. However, there are inherent differences between mice, rats, and humans in their sensory ability, along with anatomical and functional variations at key aspects of sensory processing.

**Figure 2:**
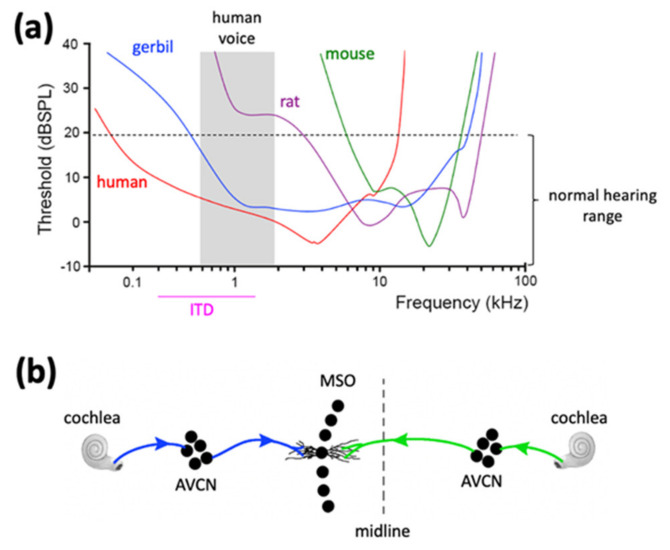
Human-like hearing ability and auditory brainstem circuit in Mongolian gerbils. (**a**) Rodent and human audiograms. Gerbils (blue), but not mice (green) or rats (purple), have good (low thresholds) low-frequency hearing, comparable to humans (red). The shaded area is the frequency range for the human voice. Threshold < 20 dB is usually considered normal hearing. Humans use low-frequency sounds (below 1500 Hz) for ITD computation. The figure is built upon published studies [98,99,107,130,131]. (**b**) The schematic shows the MSO circuit for ITD computation, which is conserved across vertebrate species that are sensitive to low-frequency sounds. As an underlying substrate of this function, bipolar dendrites of MSO neurons receive segregated inputs from the ipsilateral (blue lines) and contralateral (green lines) ears through the AVCN. Abbreviations: ITD, interaural time difference; MSO, medial superior olive; AVCN, anteroventral cochlear nucleus; FMRP, Fragile X mental retardation protein.

One marked example lies in the auditory system. Hearing is most sensitive for humans at frequencies below 4 kHz and for mice at 16 kHz (Figure 2a) [98,99]. Particularly, the normal human voice range is roughly 100 Hz–2 kHz [100], which is a frequency range that mice barely hear and rats have a high threshold for (shaded area in Figure 2a). Humans also use this low frequency range for computing interaural time differences (ITDs) [101,102], a main binaural cue for sound segregation including speech recognition in noise. Given the central role of sound segregation and vocal communication in language development and social interaction, understanding how FMRP loss impairs neuronal processing of low frequency sounds is expected to provide an important and likely necessary avenue for understanding FXS pathology. Thus, there is a critical need for additional animal models for examining whether, and if so how, deficits in processing low-frequency sounds underlie auditory dysfunction and poor language development in FXS.

A gerbil model of FXS is expected to help fill this important gap. Along with mice and rats, Mongolian gerbils (*Meriones unguiculatus*) belong to the Muridae family of rodentia [103]. Upon splitting with lineage leading to mice and rats roughly 20 million years ago [104,105], gerbils have developed certain specializations that make them of interest in sensory neuroscience. In the auditory system, gerbils have a large inner ear, which is related to good low-frequency hearing, and their hearing range covers most of the human audiogram (Figure 2a) [106,107]. In the brain, gerbils have a well-developed medial superior olive (MSO) (Figure 2b), the first auditory cell center specialized for binaural hearing using low frequency sounds [108,109]. In contrast, the MSO in mice is small [110,111], consistent with their low sensitivity to low-frequency sounds. Importantly, healthy humans have an enormous and well-organized MSO [112,113], consistent with the importance of ITD computation and low-frequency processing. With these human-like features, the Mongolian gerbil is one of the most studied experimental systems for auditory research, with an abundance of basic psychoacoustical data available [114,115] along with a huge foundation of knowledge on the structure and physiological properties of auditory neurons including MSO neurons; to name a few [116,117,118,119,120,121,122,123,124,125]. Such knowledge provides the foundation for accurately assessing auditory deficits under pathological conditions such as FXS. The MSO circuit offers a particularly attractive location in the brain for this study. For example, MSO neurons exhibit a high level of dendritic FMRP in both humans and gerbils [126]. Postmortem examination of FXS brains revealed significant MSO dysmorphology including reduced neuron numbers and abnormal neuron morphology [127,128,129].

Mongolian gerbils also have unique features in the visual system that make them an advantageous model for studies of vision under healthy and FXS conditions. Like humans, gerbils are primarily diurnal. As compared to nocturnal mice and rats, gerbils have superior acuity and better photopic vision [132,133]. The gerbil retina has a higher percentage of cone photoreceptors (13%) as compared to mice and rats (1–3%) [134,135]. Gerbils have been used to study the development and physiology of the retina [136,137] and have served as a model for understanding and treating retina pathological conditions including degenerative diseases, infections, and other types of damage [138,139,140,141]. Although there is no study of FMRP in the gerbil retina yet, FMRP expression has been documented in the developing and mature retina of humans, mice, rats, and chickens [142,143,144]. In the brain, FMRP is expressed differentially along the visual pathways of NHP and mice [145,146]. Under pathological conditions, FXS individuals exhibit macular dysplasia [147] and impaired functions for processing stimulus movement information [145]. Remarkably, FMRP level is tightly associated with visual temporal performance among healthy individuals [148] and FMR1 premutation carriers [149]. Studies of Fmr1 KO mice further demonstrated reduced retina signal transmission and retina perception [150,151] and impaired visual circuit organization and information processing in the midbrain [150,152] in the absence of FMRP.

In addition to sensory processing, gerbils provide an advantageous model for understanding social communication deficits in FXS. Mongolian gerbils have well-developed social structures and distinctive behavioral characteristics that are not observed in mice and rats. For example, like humans, gerbils establish monogamous breeding pairs and display a clear influence of paternal parenting on pup development [153]. Consistently, there is evidence that individual housing impairs gerbils’ social behavior [154,155]. Additionally, gerbils explore frequently the open arms of a radial maze following repeated exposures, a behavior not observed in mice and rats [156]. Therefore, the gerbil may serve as a suitable model for exploring genetic and molecular mechanisms of social and other behaviors that more closely resemble human conditions than mice and rats.

Metabolic studies of FXS could be another area that Mongolian gerbils could serve as an experimental model. In Fmr1 knockout mice, mitochondrial alterations have been repeatedly reported, including altered expression of mitochondrial genes, impaired mitochondrial fusion, increased oxidative stress, and reduced ATP production [157,158,159,160,161,162]. Mongolian gerbils have a greater capacity of temperature regulation and a higher metabolic rate than other rodents including mice and rats [163] and have been a popular model for studying thermoregulation under various conditions (e.g., [164,165]) and oxidative stress-mediated cerebral ischemia (reviewed in [166]). They are also used for understanding salt/water homeostasis [167] and vitamin A biosynthesis [168]. Interestingly, an association between thermoregulatory grooming and social behaviors was reported in this species [169,170].

Finally, gerbils are well suited to study epilepsy (seizures), another common pathological condition in FXS. Hyperactivity and seizures are common in FXS [9]. Approximately 10–25% of children with FXS exhibit seizures [89,171,172]. Gerbils are known to have high susceptibility to seizures [173,174]. Studies in gerbils have identified multiple seizure-inducing mechanisms including GABAergic-dependent synaptic transmission [175,176,177]. Given that seizures during early life can result in long-lasting cognitive impairments in Fmr1 KO mice [178], gerbil models of FXS have a high potential to help understand seizure generation in FXS children and further assess the impact of seizures on other cognitive and behavioral phenotypes of FXS.

The technical feasibility of developing gerbil models of FXS has been greatly enhanced with recent advancements in gerbil genome sequencing and genetic-editing tool development. CRISPR/Cas9-mediated zygote genome editing via assisted reproductive techniques enables highly efficient production of mammalian KO animals without establishing ESC lines and comprehensive genetic analyses [179,180]. Completion of the sequencing and initial annotation of the gerbil genome [181,182] would help eliminate possible off-targets while designing CRISPR/Cas9 constructs. Comparative sequencing analyses also demonstrated a high degree of similarity genome-wide and with Fmr1 specifically between the gerbil, mouse, and human [182]. Although the development of a Fmr1 KO gerbil is still on the way, CRISPR/Cas9-mediated gerbil KO strains for several other genes have recently been developed [183]. Altogether, these lines of work promise the feasibility of producing and validating Fmr1 KO gerbils.

In conclusion, Mongolian gerbils have excellent sensitivity to low-frequency sounds that are used for speech perception, superior visual perception with a high level of cone composition in the retina, and complex social architecture and behaviors as compared to mice and rats. These more human-like characteristics render the use of gerbils for more closely recapitulating the sensory and communication deficits observed in FXS humans, particularly those associated with auditory and visual information processing and those mediating social-based behaviors. Gerbils are also suited to study epilepsy and metabolic deficits, common conditions associated with FXS. Experimentally, gerbils are diurnal, quickly bred, and are easy to handle in captivity. Upon the availability of FXS gerbils, auditory brain circuits could provide precise morphological and functional assessments of FMRP loss, and auditory deficits are relatively easy to assess behaviorally, a big advantage over more complex learning or cognitive tasks. With this advantage, therapeutic approaches can be rigorously tested. Therefore, a disease model of FXS in gerbils promises to bring new insight into understanding FXS pathology and can be easily adopted for basic research and preclinical therapeutic tests.

## 4. Chicken Embryos Are a Useful Model Organism for In-Depth Dissection of FMRP Function in Assembling Neural Circuits and for Identification of the Emergence of FXS Neuropathology

Underappreciation of developmental mechanisms is another critical knowledge gap in the field of neurodevelopmental disorders. As the most frequent monogenetic form of autism and intellectual disability, FXS is at the frontier of efforts for drug development in treating neurodevelopmental disorders. In normal brains, FMRP is an RNA binding protein that provides spatiotemporal control of RNA trafficking and protein translation in dendrites and axons [184], an essential function for synaptic development and circuit assembly [185]. From studying Fmr1 KO animal models, much is known about cellular and molecular alterations in the brain at adolescent and adult ages when behavioral deficits are evident. This includes but is not limited to altered cellular and synaptic proteomes, impaired postsynaptic plasticity, and subtle but significant morphological changes in dendrites and axons [9]. Although it is commonly assumed that many deficits in FXS are derived from development, we know very little about how these deficits are generated. Similarly, very little effort has been made to prevent the initial generation of such deficits at young ages when the brain is less damaged. This lack of knowledge is very significant given the recent discovery in invertebrates that the requirement of FMRP expression for normal brain function is tightly restricted to an early developmental period [186,187,188]. Intriguingly, FXS individuals indeed show early-onset behavioral alterations and social visual engagement starting in infancy [9,189]. Thus, determining FMRP function during the embryonic stages of circuit development in vertebrates will be the beginning of a deeper understanding of FXS neuropathology.

The chicken is one of the most versatile experimental systems available for life sciences [189]. Specifically, chicken embryos provide a model organism with unique benefits for developmental studies of FXS at the molecular and cellular levels. First, both chickens and humans are precocious, meaning that a large portion of brain developmental events take place during embryonic stages. Neuron birth, differentiation, and migration at early stages are followed by neural circuit assembly during the late embryonic stage (the third trimester of human gestation). This is in contrast to rodents, in which massive synaptic connections are established after birth during the first several postnatal weeks. This difference could be highly relevant as birth-associated events could play significant roles in autism pathogenesis (reviewed in [190]). Second, easy access to chicken embryos in ovo allows site-specific genetic manipulations with a high degree of spatial and temporal control, an ability that has not been achieved by in utero manipulations in mammals. Combined with temporal control using drug-inducible vector systems, this approach reduces potential accumulating and compensatory effects during development as well as transneuronal influence from interacting synaptic components, thus allowing FMRP function determination with high precision. Third, chickens possess neural circuits that are organized and function in comparable manners as mammals and yet with a less complicated brain anatomy. This allows sensitive assessment of changes in neuronal structure and properties in abnormal brain development without losing clinical relevance to human diseases.

The chicken homolog of the *FMR1* gene was first cloned and characterized in 1996 [191]. The nucleotide and amino acid sequences of chicken FMRP are remarkably similar to human FMRP (85% and 92% identities, respectively), with identical phosphorylation and mRNA binding sites [126,191]. Indeed, the chicken FMRP binds a subset of brain mRNAs including its own mRNA [191,192], and the binding motif of *FMR1* gene is conserved between human and chicken [193]. Recently, genome-wide molecular characterizations of chickens from embryonic to adult stages became available [194], further enhancing the broadness and depth of genetic studies in chickens.

Studies of chicken embryos following targeted FMRP misexpression have uncovered a number of novel FMRP functions in developing auditory neurons and circuits. It is well-established that auditory hindbrain assembly shares the same developmental framework between birds and mammals [195,196]. Chickens are low-frequency listeners, within a hearing frequency range of up to 4–5 kHz. The two key excitatory nuclei of the chicken auditory brainstem, the nucleus magnocellularis (NM) and nucleus laminaris (NL), are structurally and functionally similar to the mammalian anteroventral cochlear nucleus and MSO, respectively. NM neurons receive temporally locked excitation from the auditory ganglion neurons in the inner ear, and in turn, send bilaterally segregated signals to the NL. Like the mammalian MSO, bipolar neurons in the avian NL are specialized to compute ITD for sound localization and segregation [197,198]. Structure and physiological properties of developing and mature NM and NL neurons are well-characterized (e.g., [199,200,201,202,203,204,205,206,207,208,209,210]), providing an enormous advantage for designing experiments and interpreting results.

**Figure 3:**
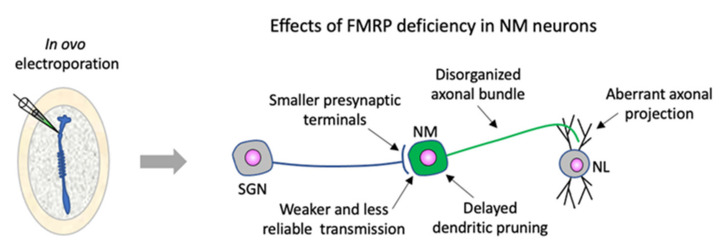
Site-specific FMRP functions in the chicken NM circuit. In ovo electroporation of FMRP-shRNA or CRISPR-mediated KO constructs into NM precursor cells at embryonic day 2 (left). Five distinct phenotypes were observed at subcellular levels later in development during axon navigation and circuit assembly (right). Abbreviations: SGN, spiral ganglion neurons; NM, nucleus magnocellularis; NL, nucleus laminaris; FMRP, Fragile X mental retardation protein.

The journey for understanding FMRP function in the NM–NL circuit began with the identification of intensive FMRP expression in NM and NL neurons [126]. Proteomic analyses of tissue samples collected specifically from these auditory cell groups provided a list of putative mRNA targets of FMRP relevant to NM and NL neurons [192]. Subsequently, FMRP actions during NM and NL development were examined in vivo [211,212], adopting genetic approaches using drug-inducible constructs that affect the target throughout development or during specific time windows [213]. Specifically, drug-inducible Fmr1-shRNA constructs were delivered via in ovo electroporation into NM precursor cells at an early embryonic stage, producing a mosaicism of FMRP expression in NM neurons, with reduced and normal FMRP levels in neighboring transfected and nontransfected neurons, respectively. This pattern allows for robust statistical analyses between FMRP-deficient and FMRP-intact neurons, axons, and terminals within the same local environment. This is very significant because it avoids individual variations that are inherent with between-animal comparisons, the predominant strategy used in studies of KO animal models. Studies on chicken embryos have made several important observations pertaining to FMRP functions (Figure 3). First, a cell-autonomous effect of FMRP deficiency on dendritic development was observed [212]. Normally, auditory nerve axons form large axosomatic endbulb synapses on the cell bodies of NM neurons. These neurons grow extensive dendrites at early stages and retract these dendrites when endbulbs begin to form. Neurons transfected with Fmr1 shRNA exhibited a remarkable delay in dendritic branch retraction, which is expected to affect dendritic integration of afferent inputs and timely formation of large endbulb terminals. Second, a transsynaptic effect was observed on the incoming auditory nerve terminals. FMRP reduction in NM neurons led to smaller presynaptic endbulbs with a reduced morphological complexity. Patch-clamp recording from FMRP-shRNA-transfected NM neurons confirmed functional consequences of dendritic and synaptic deficits on neurotransmission, showing smaller amplitudes and slower kinetics of spontaneous and evoked excitatory postsynaptic potentials. Third, examining the developmental trajectory of NM axons projecting upon NL dendrites revealed additional roles of FMRP in axon growth and projection [212]. CRISPR or shRNA-mediated FMRP KO and knockdown resulted in disorganized axonal bundling, delay in midline crossing, and aberrant axon over-projection within the NL. These effects may be associated with local acute effects of FMRP loss, an idea that is supported by the presence of FMRP-containing puncta along the developing NM axons. Thus, FMRP in NM neurons has a diverse range of functions depending on the subcellular sites of protein localization.

While they are a newly established model for FMRP and FXS research, chicken embryos have a long-standing history for studying a wide variety of neurodevelopmental disorders. These include neural tube-related diseases [214], fetal alcohol spectrum syndrome [215,216], and Joubert syndrome [217]. Chicken embryos are also used for studying drug- and virus-induced abnormal brain development [218,219]. This diversity will facilitate the identification of underlying mechanisms that are common across diseases of distinct etiology. Intriguingly, chicken embryos have been promoted for use as a pharmacology model in replace/reduce use of rodents [219]. Further advancement in this field would enable direct application of the knowledge gained from studying chicken embryos to preclinical studies.

## 5. Conclusions

New animal organisms have been adopted or are in development for FXS research and are expected to fill several important gaps that existing animal models have not filled. We anticipate that an NHP model of FXS will provide a unique series of molecular and phenotypic features and provide an exciting opportunity to address a range of neurological questions that link cellular, molecular and behavioral phenotypes in FXS and potentially other autism spectrum disorders. Modeling FXS in Mongolian gerbils is expected to more closely recapitulate the sensory and communication deficits observed in FXS, particularly those associated with auditory and visual information processing and those mediating social-based behaviors. Finally, advanced genetic and imaging approaches in chicken embryos allow in-depth characterization of FMRP functions during circuit assembly periods with high temporal and spatial resolutions and clinical relevance. These models, along with existing rodent and invertebrate models, are expected to contribute significantly to the next phase of FXS research and neurodevelopmental disorder studies.

## Data Availability

Not applicable.
